# CT chest for COVID-19, a multicenter study—experience with 220 Egyptian patients

**DOI:** 10.1186/s43055-020-00263-6

**Published:** 2020-07-31

**Authors:** Youssriah Yahia Sabri, Amr A. Nassef, Iman Mohamed Hamdy Ibrahim, Mohammed Raafat Abd El Mageed, Mostafa Ahmed Khairy

**Affiliations:** Department of Diagnostic and Interventional Radiology, Kasr Al Ainy Faculty of Medicine, Cairo University, Cairo, Egypt

**Keywords:** CT chest, COVID-19, Common patterns, Uncommon patterns, Severity score, Temporal changes, Reporting, Pitfalls

## Abstract

**Background:**

COVID-19 has become a national and an international pre-occupation to all doctors. Dealing with patients with clinical suspicion of COVID-19 is a daily markedly growing professional issue for radiologists. The number of COVID-19 cases we deal with is peaking since last March and so is our experience in recognizing the disease patterns and in assessing its severity. The purpose of this study is to assess the role of CT chest in the diagnosis of COVID-19 based on our experience with 220 Egyptian cases.

**Results:**

A cross-sectional multicenter study involving 220 patients; 68 (30.9%) females and 152 (69.1%) males, their age range was 10-92 years (average 49.198 years). Non-contrast MSCT chest was done to patients with clinically suspected COVID-19. Data assessment and analysis for lesions probability, pattern, localization, and severity were done.

Bilateral affection was seen in 168/220 cases (76.36%). Multilobar affection was noted in 186/220 cases (84.54%). Lower lobes affection was noted in 179/220 cases (81.36%). Peripheral/subpleural affection was noted in 203/220 cases (92.27%). The common CT patterns (ground-glass opacities, consolidation, crazy paving, vascular thickening, traction bronchiectasis, vacuolar sign, architectural distortion signs, and reversed halo sign) and the uncommon CT patterns (halo sign, masses, nodules, lobar affection, tree in-bud-pattern and cysts) were discussed. Associated extra-pulmonary lesions described. Temporal changes, severity scoring, reporting, and possible pitfalls were all assessed.

**Conclusion:**

In our experience, CT plays a basic essential role in diagnosing COVID-19 in the current declared pandemic.

## Background

COVID-19 is the disease caused by the coronavirus named SARS-CoV-2. It has been declared as a pandemic [1] by the WHO in March 2020. The disease has been first reported in China [2, 3] in December 2019, and since then a flood of articles discussing the disease imaging has been published and available online. The first interest was determining the role of imaging and stating the typical and atypical patterns of the disease [4–9] and many classifications have been available considering typical, atypical, indeterminate CT patterns for COVID-19, where only normal CT was considered as negative. Based on this, a step further was taken, some institutes and societies declared RAD systems namely CO-RAD and COVID-Rad. Severity scores based on CT findings were introduced with relatively limited application to the institutes’ concerned [6, 7, 10, 11].

The Egyptian ministry of health latest declaration on the 31 of May 2020, was 24,985 total COVID-19 cases with 1536 new ones (Www.care.gov.eg). COVID-19 is a national and an international pre-occupation to all doctors. As radiologists, dealing with patients with clinical suspicion of COVID-19 is a daily markedly growing professional issue. The number of cases we deal with is peaking since last March as well as our experience in dealing with recognition of the disease patterns and assessing its severity.

## Aim of the work

The aim of this study was to assess the role of CT chest in the diagnosis of COVID-19 based on our experience with 220 Egyptian cases.

## Methods

This cross-sectional study involved 220 patients: 68 (30.9%) females and 152 (69.1 %) males; their age range was 10-92 years (average 49.198 years). Three children two females and a male aged 10, 12, and 13 years old were included in this study. The study was a multicenter study done from the beginning of March to the end of May 2020. All cases were clinically suspected to suffer from COVID-19 and CT chest was requested as a part of the patients’ set of investigation. Most cases were symptomatizing (195/220 [about 89%]) with symptoms ranging from 1 day to 2 weeks’ time, while 25/220 (about 11%) were non-symptomatizing with history of contact with a COVID-19 patient. Symptoms were fever, cough, and dyspnea. Vomiting was present in two patients and headache in one patient. One hundred ten (50%) patients were known to have systemic or chronic diseases (see Table 1 for details).

**Table 1:** List of patients’ number and percent known to have systemic or chronic diseases

Disease	Number of patients	Percentage
Cardiac disease	12	5.45%
Systemic hypertension	56	25.45%
Diabetes mellitus	23	10.45 %
Liver cirrhosis	11	5%
Renal impairment	4	1.81%
Cancer breast	1	0.45%
Bronchial asthma	3	1.36%

Only patients with positive CT findings were included in this study.

Non-contrast MSCT study of the chest was done to all the patients. The studies were assessed by two radiologists with an experience in radiology ranging from 11-32 years.

### CT assessment and data analysis

The scans were performed using a Somatom, Siemens, 64-MDCT scanner, and a Philips, 256-MDCT scanner.

The scout was taken in supine position, kV 120, mA 25 during holding breath in full inspiration.

Scans were obtained during full inspiration in a supine position using the following parameters; 120 kV, 130-240 mAs, 5 mm beam collimation, 1.25 pitch, 0 gantry tilt, and the FOV depending on patient’s size. The scans covered the whole thorax from the root of the neck to below diaphragm.

No intravenous contrast administered.

Following acquisition, the acquired images were transferred to dedicated post-processing workstation and volumetric measurements were obtained by applying the multiplanar reformation function.

The points of interest in CT assessment and data analysis were as follows:

1. Assessing pulmonary lesions site and distribution regarding their laterality, lobes affected, multilobar versus single lobe affection, and distribution within the parenchyma (peripheral, patchy, central, diffuse, and lobar).
2. Assessing the patterns of lesions encountered and their prevalence, having the Fleischner society glossary of terms for thoracic imaging (2008) [12] as our main reference for identifying and naming the lesions.
3. Assessing associated extra-pulmonary chest lesions.
4. Assessing temporal changes of CT findings in our patients.
5. Surfing the literature for different available published classifications for describing the CT lesions for COVID-19, assessing severity and for reporting.

### Statistical methods and data analysis

Data was entered on the computer using the “Microsoft Office Excel Software” program (2010) for windows.

Data was then transferred to the Statistical Package of Social Science Software program, version 23 (IBM SPSS Statistics for Windows, Version 23.0. Armonk, NY: IBM Corp.) to be statistically analyzed.

Data presented using range, mean, standard deviation, median, and interquartile range for quantitative variables and frequency and percentage for qualitative ones.

## Results

This cross-sectional multi-center study involved 220 patients; 68 (30.9%) females and 152 (69.1 %) males, their age range was 10-92 years (average 49.198 years). Non-contrast MSCT of the chest of all patients was assessed by two experienced radiologists.

## Lesions distribution

### Laterality

- Bilateral affection was seen in 168/220 cases (76.36%).
- Unilateral affection was seen in 52/220 cases (23.64%) as follows**:**
- 32 cases in right lung (14.54%)
- 20 cases in left lung (9.1%)

### Lobes affection

- Multilobar affection was noted in 186/220 cases (84.54%).
- Single lobe affection was noted in 34/220 cases (15.45%) having the following:

- Single focus in 15 cases (6.81%) [13 cases {5.9%} with ground glass opacity, 2 cases {0.9%} with consolidation].
- Multiple foci in 19 cases (8.64%) [13 cases {5.9%} with ground glass opacity, 3 cases {1.36%} with consolidation, and 3 cases {1.35} with masses and nodules].

Table 2 shows details of lobes affection.

**Table 2:** Lobar affection

Lobe affected	Number of patients	Percentage
Upper lobe	125	56.82%
Lower lobe	179	81.36%
Middle lobe	98	44.54%
Lingula	122	55.45%

### Distribution of lesions in parenchyma

Table 3 shows the distribution of lesions in parenchyma.

**Table 3:** The distribution of lesions in parenchyma

Distribution	Number of patients	Percentage
Peripheral/subpleural	203/220	92.27%
Patchy	118/220	53.64%
Central	34/220	19.1%
Lobar	42/220	15.45%
Diffuse	0/220	0%

## Patterns of lesions encountered

### Common lesions

#### Ground glass opacities (Figs. 1, 2, 3, 4, 5, 6)

- This pattern was encountered in 194/220 cases (88.16%).
- It was mostly subpleural/peripheral in location (92.27%) with vascular thickening noted within the lesions or in their vicinity.
- Out of the 34 cases with single lobe affection, 26 showed ground glass opacities (76.5%), 13 of whom had a single ground glass opacity lesion.

**Fig. 1:**
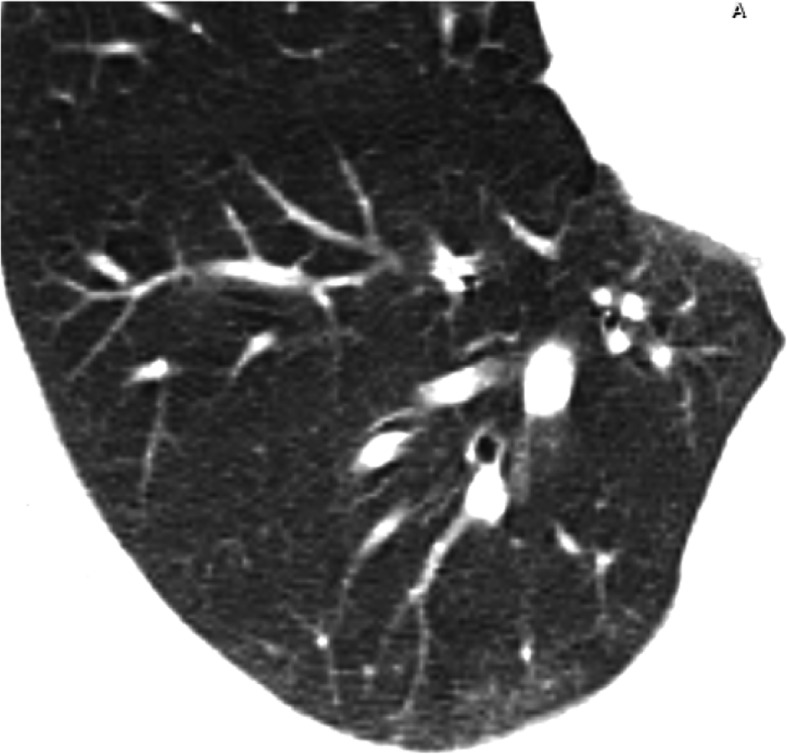
Male patient 38 years old with COVID-19. Magnified axial CT image shows right lower lobe subpleural ground glass opacification with prominent vessels within

**Fig. 2:**
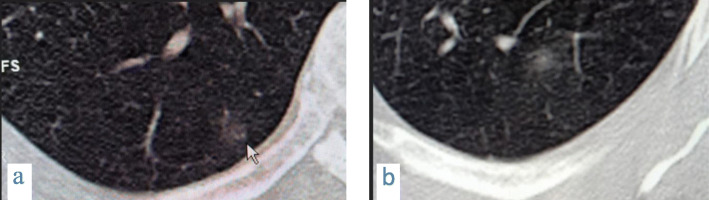
A 56-year-old male patient with COVID-19, CT chest magnified axial images showing (**a**) small fairly rounded subpleural ground glass opacity in the right lower lobe (arrow) and (**b**) a left lower lobe fairly rounded peripheral small ground glass opacity

**Fig. 3:**
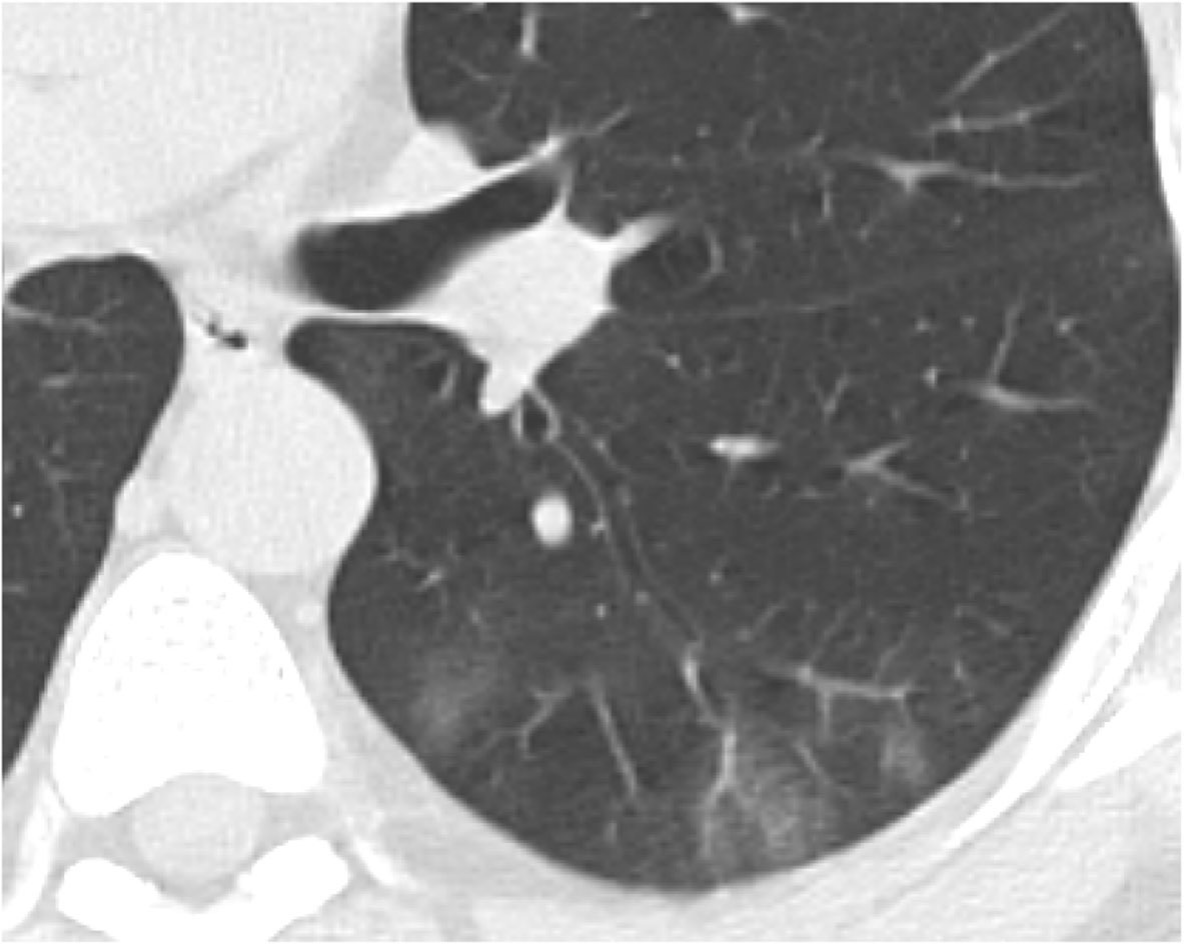
Male patient 43 years old with COVID-19. Magnified axial CT image shows left lower lobe subpleural ground glass opacifications with vascular thickening within and traction bronchiectasis

**Fig. 4:**
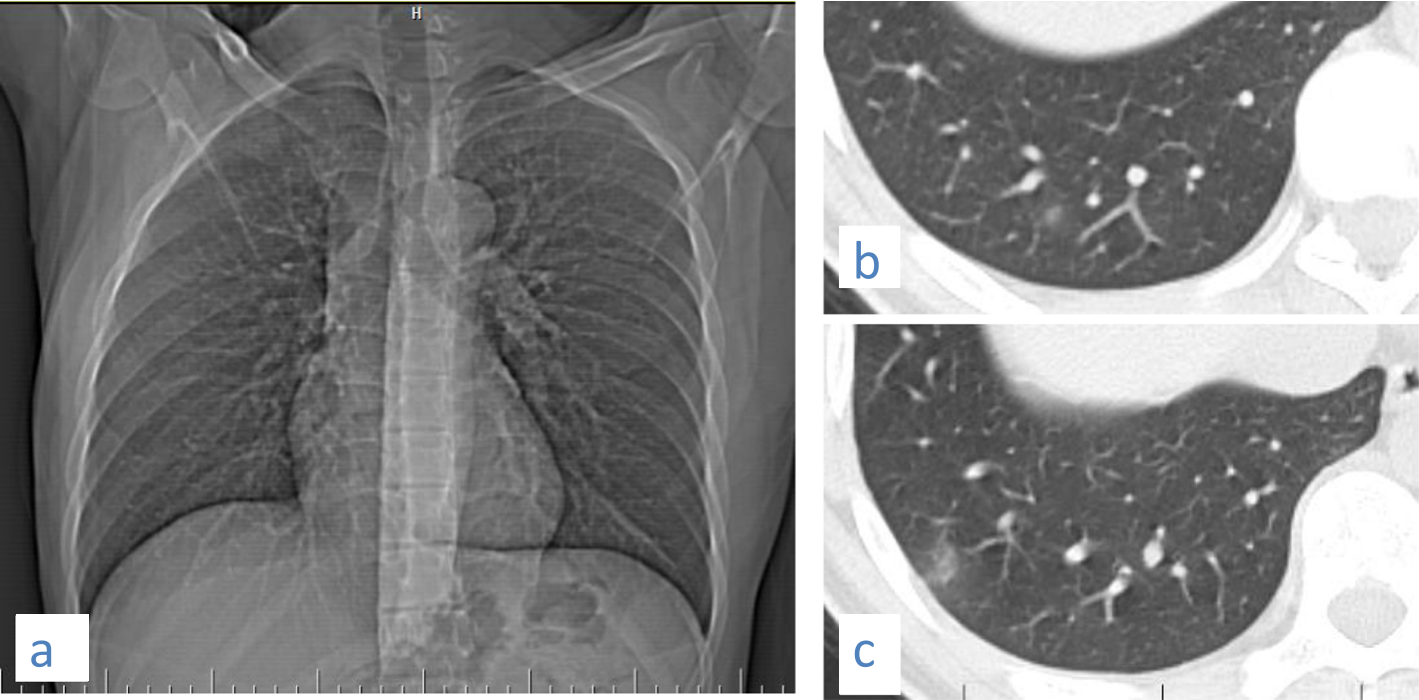
Male patient 28 years old with COVID-19. CT scout image (**a**) shows no abnormality while magnified axial CT image (**b**) shows right lower lobe peripheral rounded ground glass opacity, and (**c**) shows a subpleural nodule

**Fig. 5:**
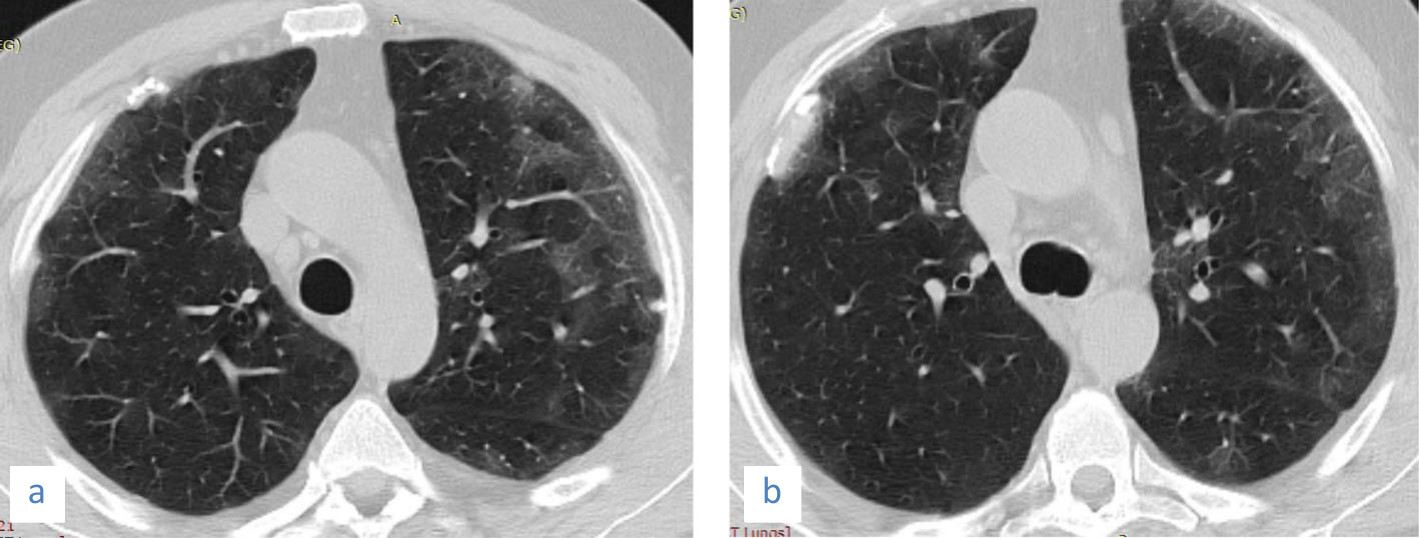
Male patient 56 years old with COVID-19. CT axial images (**a**) and (**b**) show bilateral predominantly peripheral rounded ground glass opacities with vascular thickening. Note the bilateral pleural plaques with calcification due to asbestos exposure

**Fig. 6:**
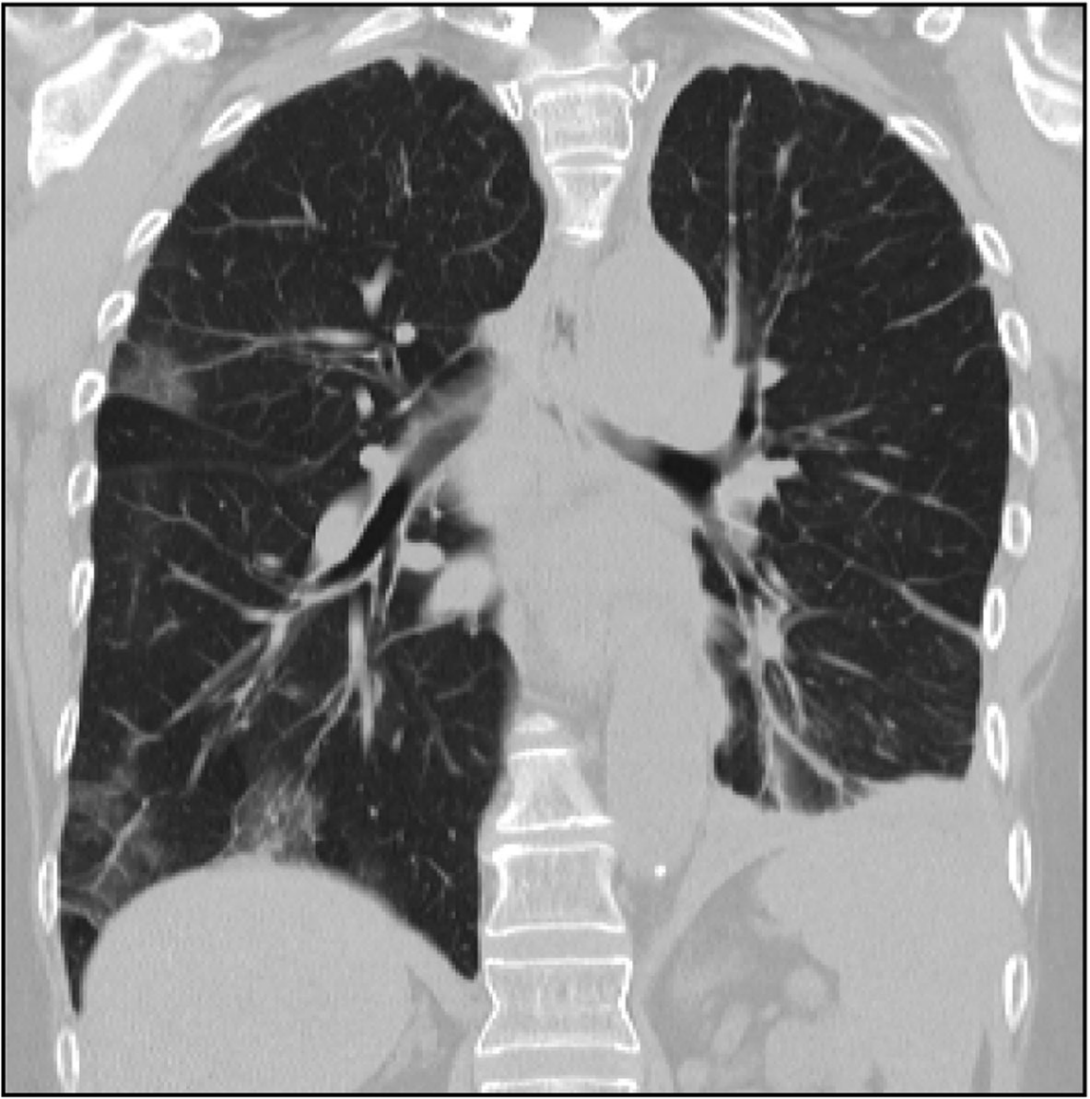
Female patient 70 years old with COVID-19. CT coronal image shows peripheral ground glass opacities with vascular thickening in the right lung. Note the left mild pleural effusion

#### Consolidation (Figs. 7, 8)

- This pattern was encountered in 140/220 cases (66.7%).
- It was mostly subpleural/peripheral in location (74.75%); however, lobar (15.45%) and central (10%) distribution was noted.
- Traction bronchiectasis was seen related to consolidation in 72 cases (32.72%), while air-bronchogram was seen in 86 cases (39.1%).

**Fig. 7:**
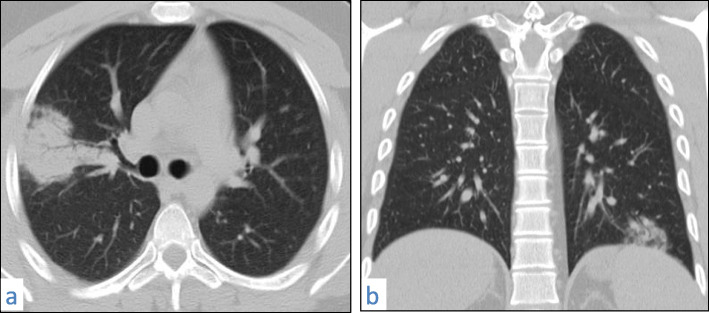
A 31-year-old male patient with COVID-19, CT chest axial image (**a**) shows right upper lobe predominantly subpleural consolidation with air bronchogram, coronal image (**b**) shows left lower lobe subpleural consolidation with traction bronchiectasis and a tiny lucency “vacuolar sign”

**Fig. 8:**
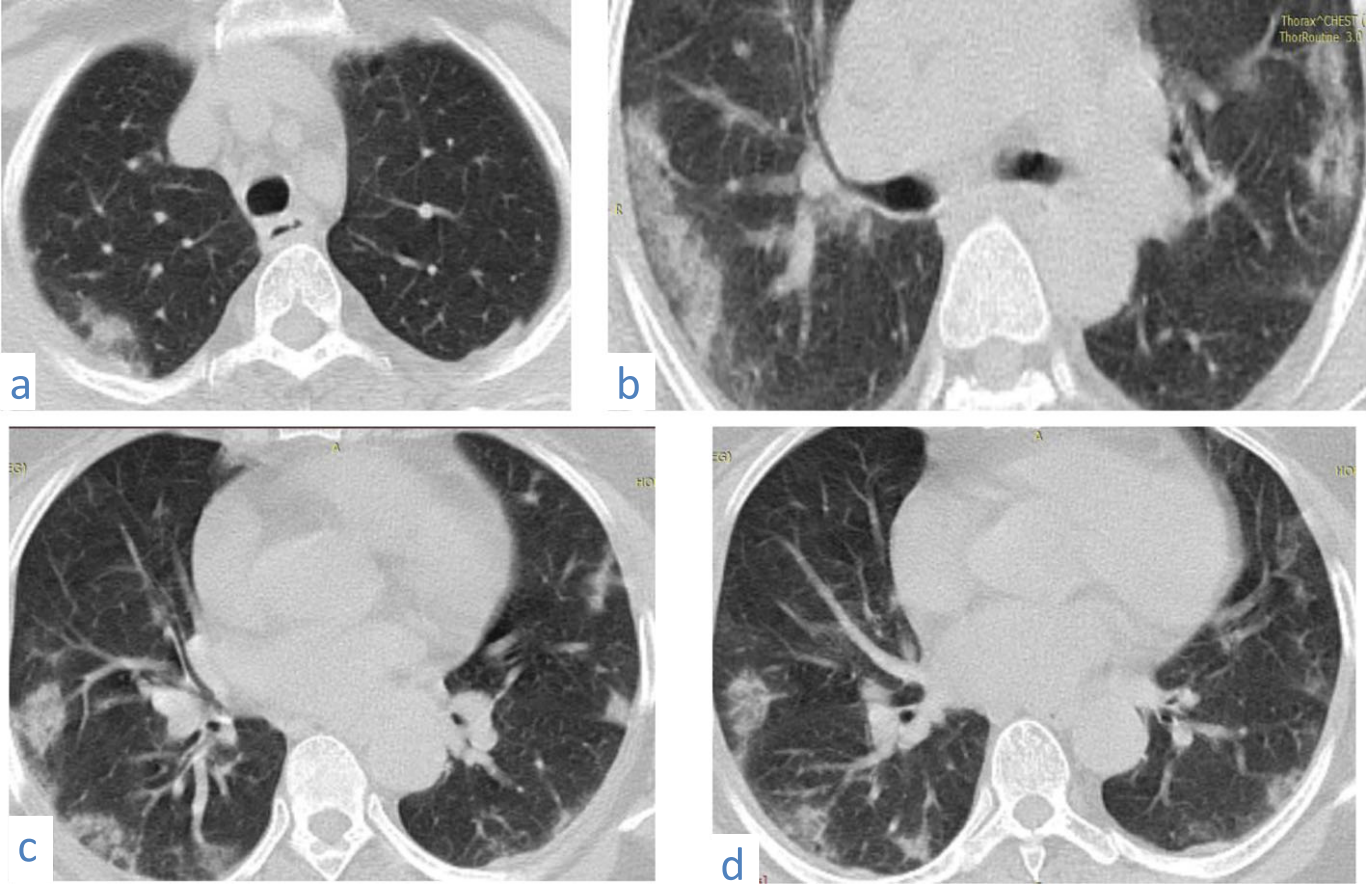
A 57-year-old male patient with COVID-19, CT chest axial images (**a**–**d**) show bilateral predominantly subpleural consolidation with tiny lucencies within “vacuolar sign”

#### Crazy-paving pattern (Figs. 9, 10, 11)

- In this pattern, ground-glass opacity shows superimposed thickened interlobular septa and intralobular lines.
- This pattern was encountered in 82/220 cases (37.27%). It was mostly subpleural/peripheral in location with vascular thickening noted within the lesions.

**Fig. 9:**
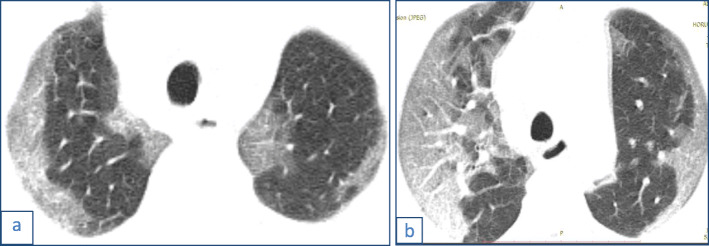
A 53-year-old male patient with COVID-19, CT chest axial images (**a**) and (**b**) show subpleural crazy paving of all lobes with vascular thickening within

**Fig. 10:**
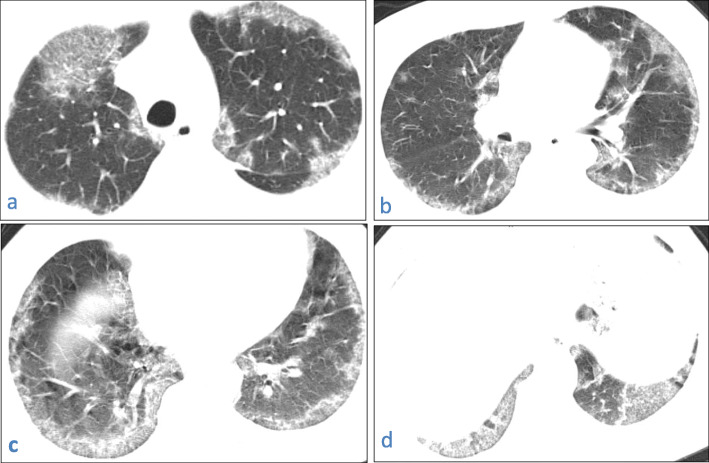
A 67-year-old male patient with COVID-19, CT chest axial images (**a**-**d**) show predominantly subpleural crazy paving of all lobes with vascular thickening within. The affection is mostly in both lower lobes

**Fig. 11:**
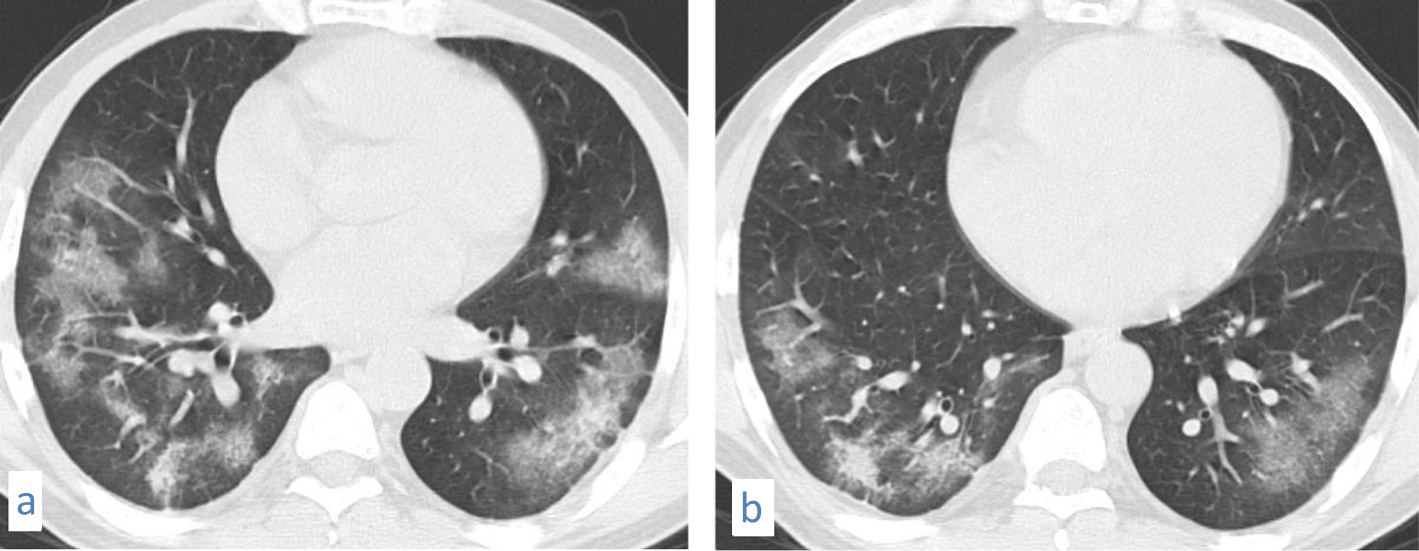
A 37-year-old male patient with COVID-19, CT chest axial images (**a**) and (**b**) show predominantly peripheral crazy paving with vascular thickening and traction bronchiectasis within. The affection is mostly in both lower lobes. A right peripheral basal patch of consolidation is noted

#### Vascular thickening

- Was seen in all of our patients (100%) either within a lesion or in its vicinity.
- It is most clearly seen related to ground glass opacities and crazy-paving (Figs. 1, 3, 5, 6, 7, 8, 9, 10, 11).

#### Traction bronchiectasis

- Was seen in 142/220 patients (about 64.54%) either within or in the vicinity of a lesion (Figs. 3, 7, and 11).

#### Vacuolar sign

- Was seen in 87/220 patients (about 39.54%) seen as lucent locule(s) within a lesion; consolidation, mass, ground glass opacity, or crazy-paving (Figs. 7, 8, and 19).

#### Signs of architectural distortion (Figs. 12, 13, 14, 15)

- Was seen in 82/220 patients (about 37.27%).
- Many signs have been described in literature [13, 14] all reflecting fibrosis and architectural distortion, of which the perilobular fibrosis that usually shows as arcade–like sign, was noted in 67/82of our cases (81.70%). Subpleural bands and fibrous stripes were seen in 33/82 cases (40.24%). Spider web pattern was seen in 12/82cases (14.63%).

**Fig. 12:**
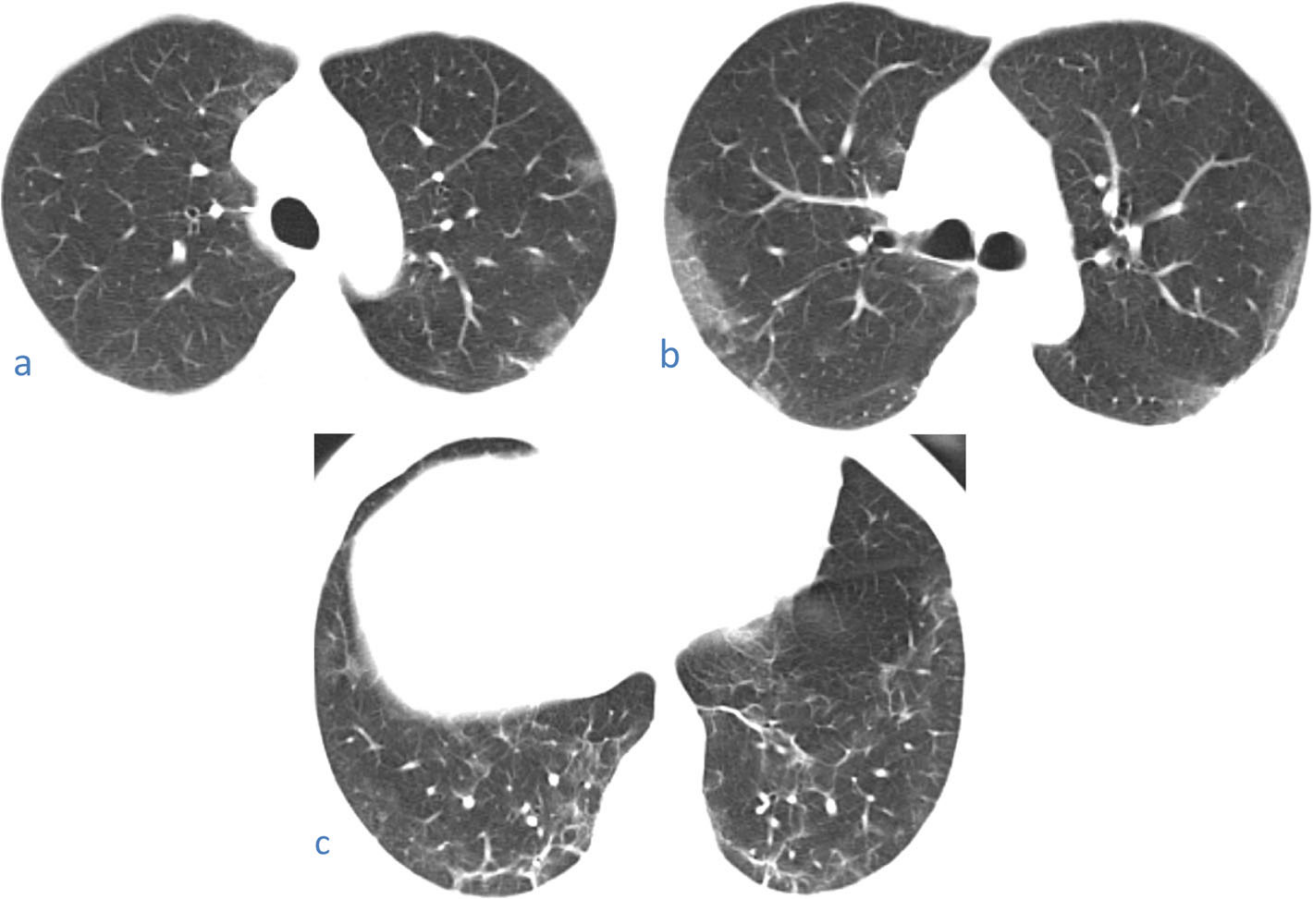
A 50-year-old female patient with COVID-19, known patient of bronchial asthma presented with 10 days of fever, CT chest axial image (**a**) shows left upper lobe subpleural ground glass opacities with vascular thickening (**b**) shows bilateral subpleural crazy paving and (**c**) shows bilateral basal subpleural arcade opacities; perilobular thickening and mild architectural distortion

**Fig. 13:**
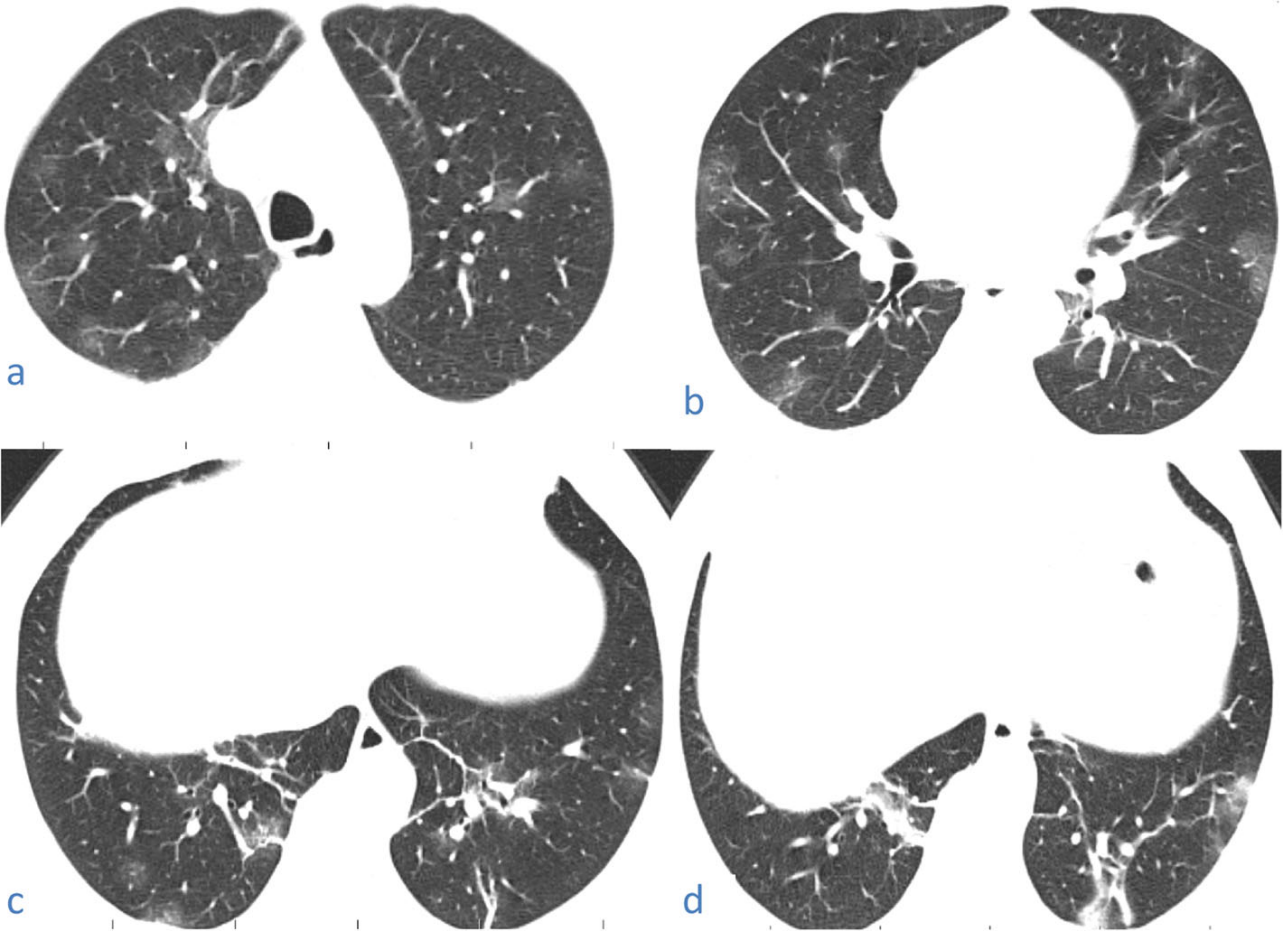
A 40-year-old male patient with COVID-19. CT chest axial images (**a**) and (**b**) show subpleural and patchy ground glass opacities with vascular thickening, (**c**) and (**d**) show bilateral basal subpleural areas of ground glass opacities and consolidation with fibrous stripes, architectural distortion, spider web opacities, and perilobular thickening

**Fig. 14:**
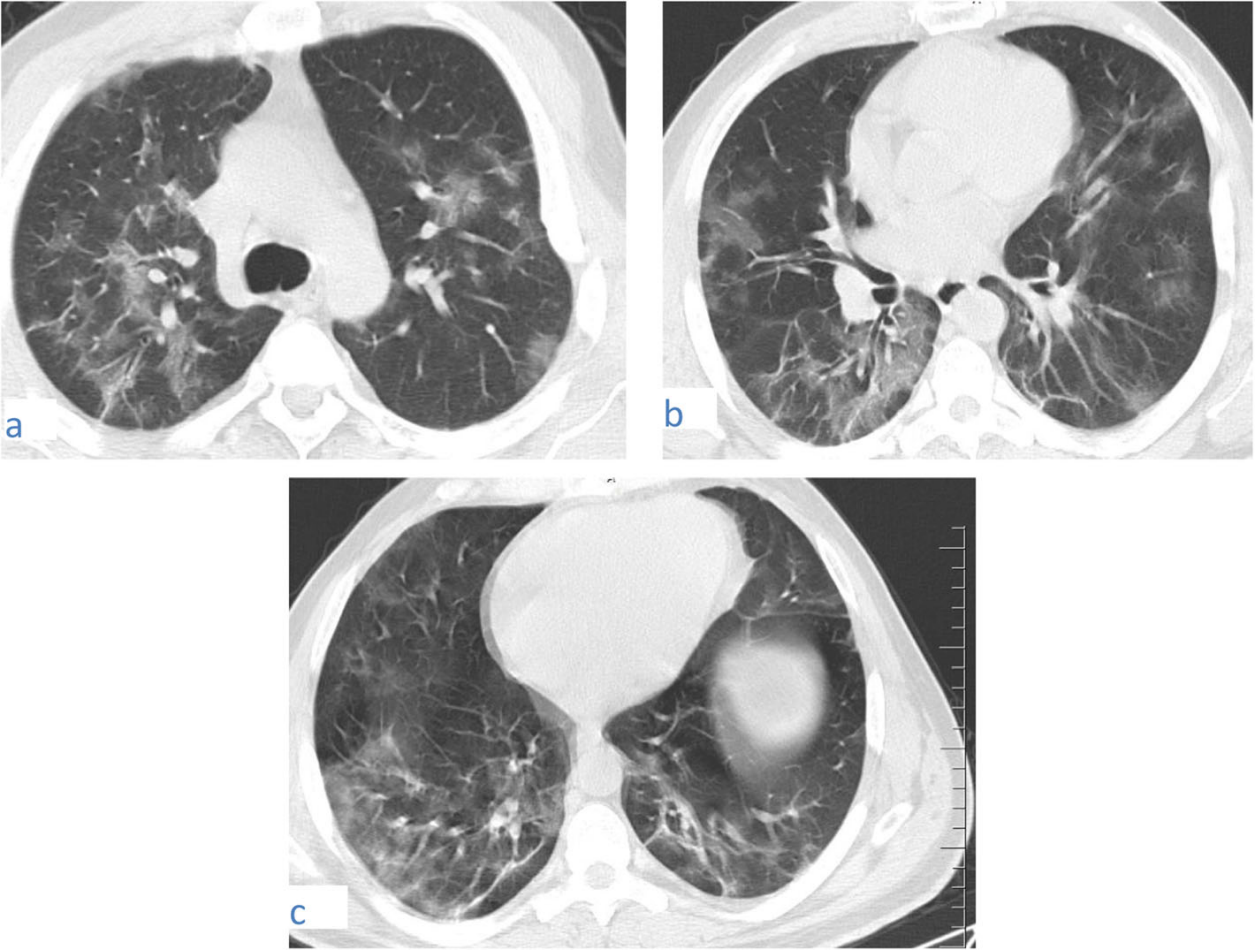
A 38-year-old male patient with COVID-19. CT chest axial images (**a**), (**b**), and (**c**) show subpleural and patchy ground glass opacities with vascular thickening, and show bilateral basal subpleural areas of ground glass opacities, fibrous stripes, architectural distortion, and perilobular thickening

**Fig. 15:**
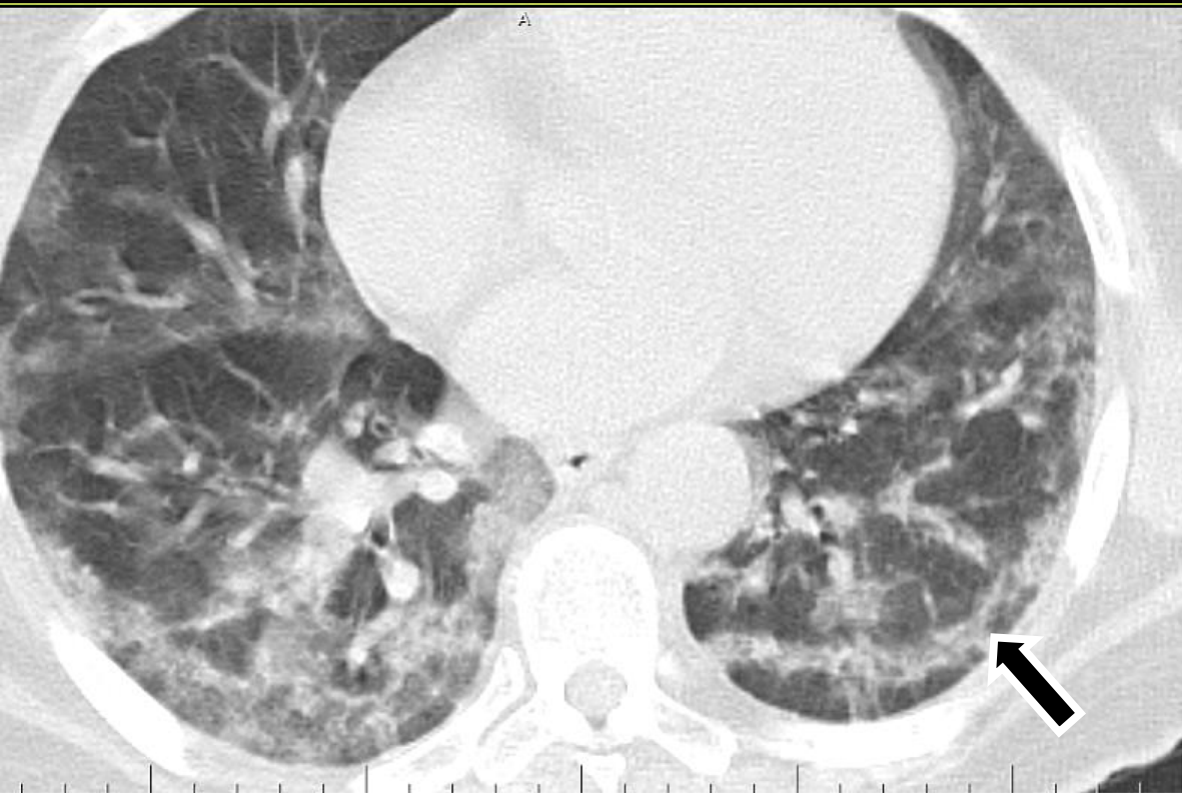
A 60-year-old female patient with COVID-19. CT chest axial image shows bilateral subpleural crazy paving, architectural distortion, and perilobular thickening. A subpleural band is seen on the left (black arrow)

#### Reversed halo sign

**Fig. 16:**
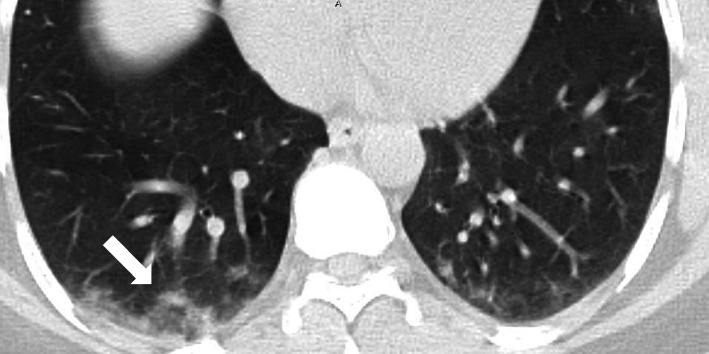
A 37-year-old female patient with COVID-19 with 4 days of fever cough and dyspnea, CT chest axial image shows predominantly subpleural ground glass opacities with vascular thickening and shows a right lower lobe subpleural opacity with “reversed halo” sign (arrow)

- Was seen in 63/220 patients (about 29%) seen as a focal fairly rounded area of ground glass opacification surrounded by a near complete ring of consolidation (Figs. 16 and 18).

### Uncommon lesions (Figs. 17, 18, 19, 20)

Table 4 shows a summary of uncommon lesions encountered in our study.

**Fig. 17:**
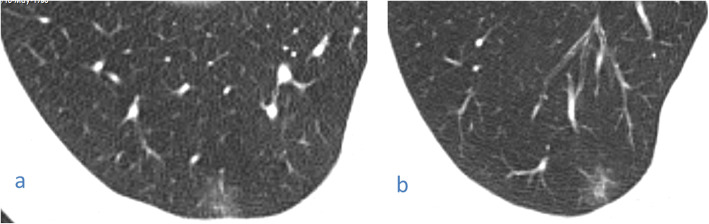
A 60-year-old male patient with COVID-19, CT chest axial image (**a**) shows subpleural ground glass opacity with vascular thickening and (**b**) shows a subpleural nodule with “halo” sign

**Fig. 18:**
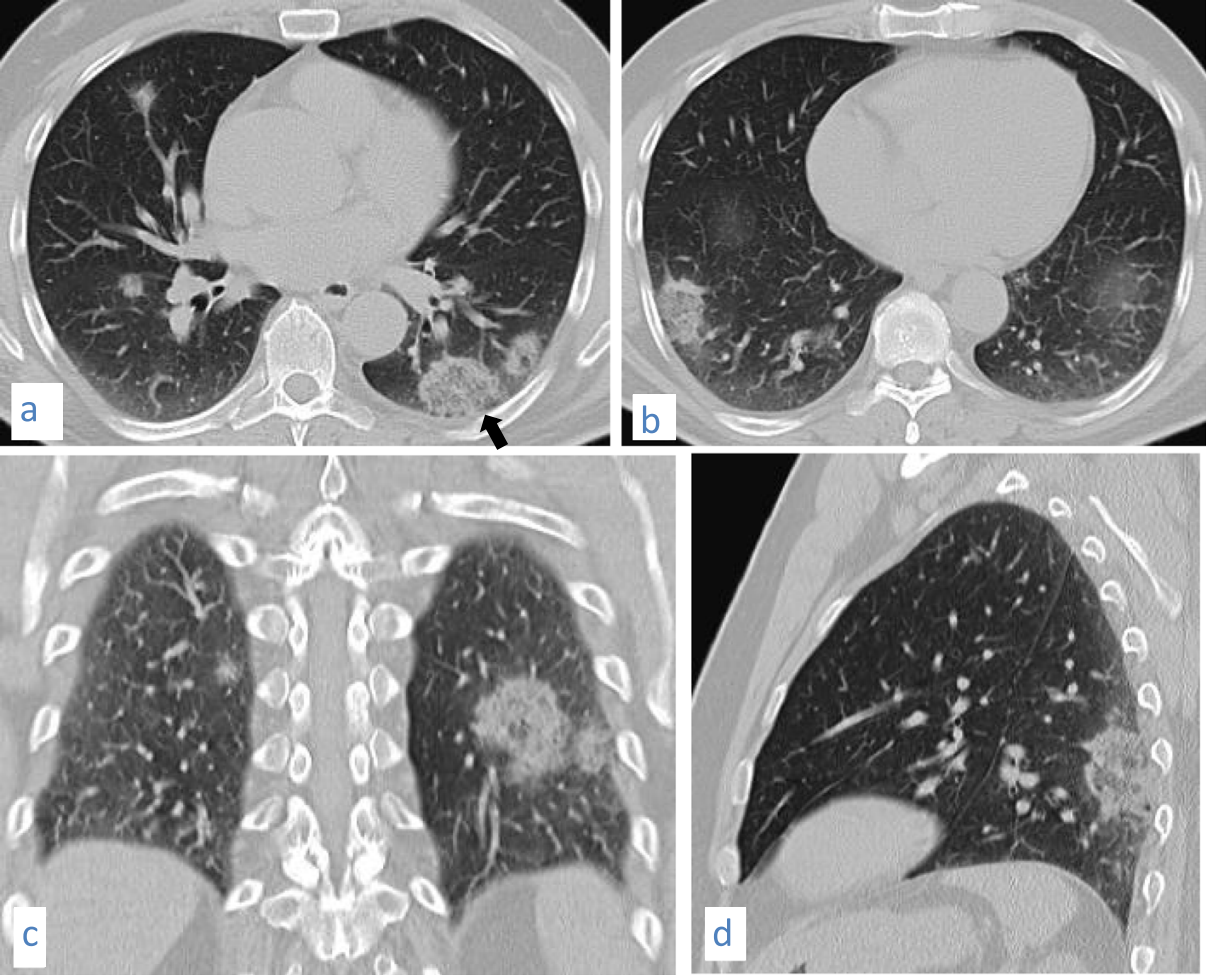
A 53-year-old male patient with COVID-19. CT chest axial images (**a**) and (**b**), coronal image (**c**), and sagittal image (**d**) show subpleural ground glass opacities with vascular thickening, nodules and masses some show “reversed halo” sign (arrow)

**Fig. 19:**
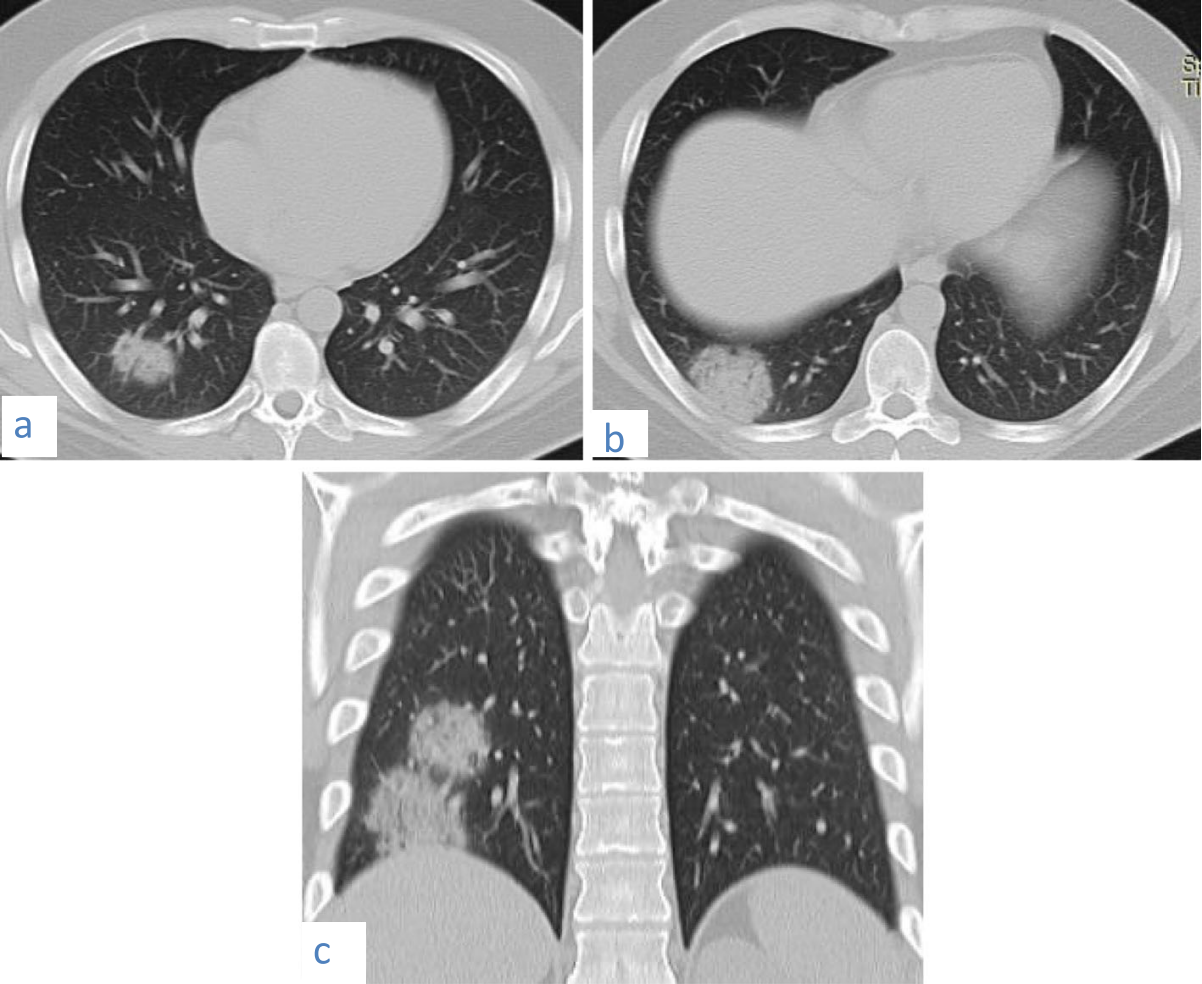
A 27-year-old male patient with COVID-19. CT chest axial images (**a**) and (**b**) and coronal image (**c**) show two subpleural masses showing “vacuolar” sign

**Fig. 20:**
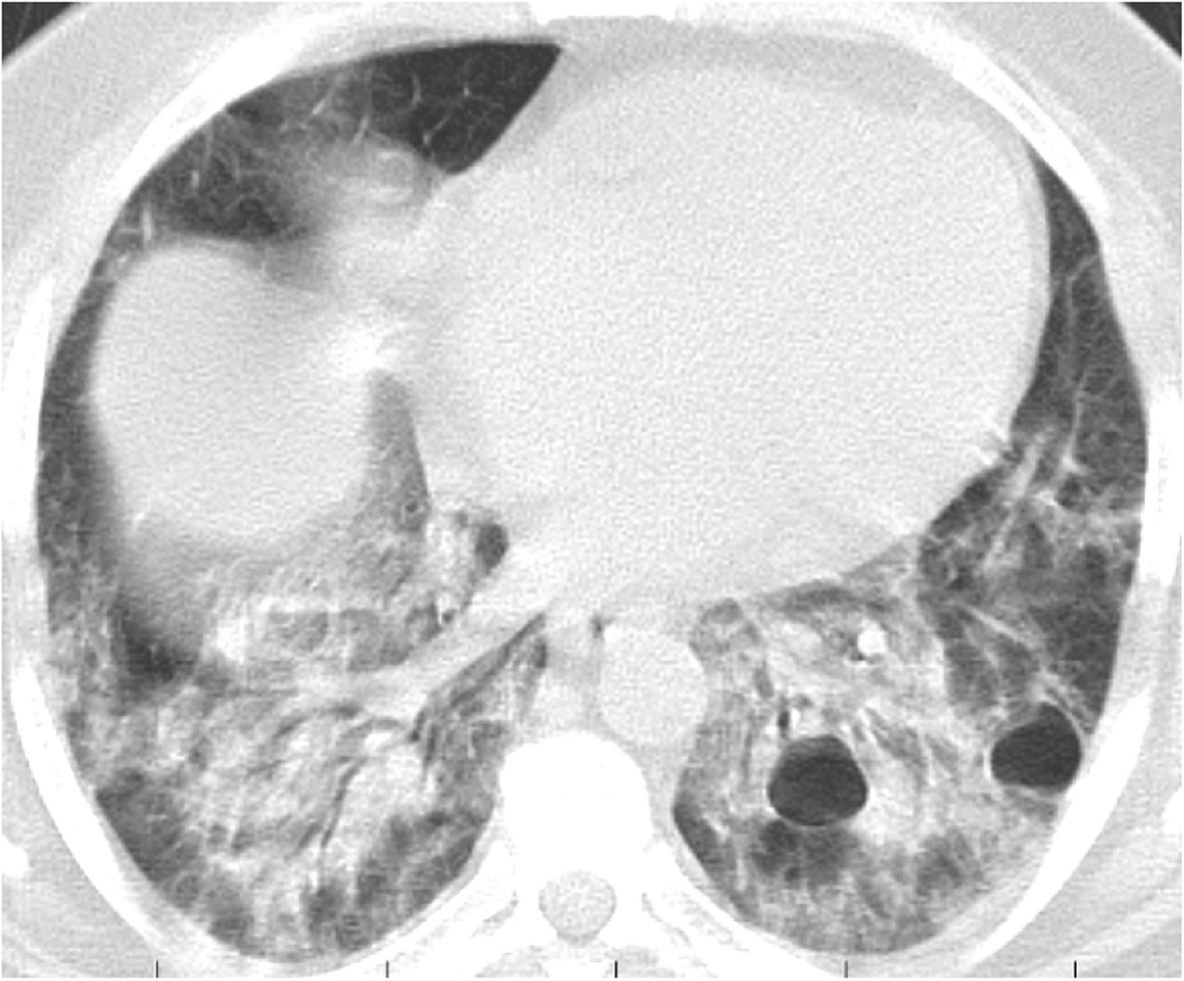
A 50-year-old male patient with COVID-19. The patient is a known hypertensive and diabetic. CT chest axial image shows bilateral extensive consolidation of both lower lobes with air-bronchogram. Two air–containing lungs cysts are seen in the left lower lobe lesion

**Table 4:** Summary of uncommon lesions

Type of lesion	Number of patients	Percentage
Nodules	12	5.45%
Masses	5	2.27%
Prominent interlobular septa	3	1.36%
Centrilobular nodules and tree-in bud pattern	7	3.2%
Air-containing cysts	3	1.36%
Halo sign	6	2.72%

### Extra-pulmonary lesions

#### Pleural lesions

- Posterior multifocal mostly basal trivial pleural thickening was seen in 145/220 cases (66%).
- Pleural effusion was seen in 13/220 cases (6%); mild in 9 cases (about 4.1%), moderate in 4 cases (1.81%), bilateral in 8 cases (3.63%), and unilateral in 5 cases (2.27%).
- Loculated right lateral hydropneumothorax was seen in one case (0.45%).
- Bilateral pleural plaques with foci of calcification were seen in one case with associated asbestos exposure (Fig. 5).

#### Esophageal lesions

- Was seen in 90/220 patients (about 40.9%).

Table 5 shows a summary of esophageal lesions encountered in our study.

**Table 5:** Summary of esophageal lesions

Type of lesion	Number of patients	Percentage
Hiatus hernia	48	21.81%
Rather patulous esophagus ( mostly lower thoracic)	40	18.18%
Fluid retained in upper esophagus	1	0.45%
Esophageal wall thickening	1	0.45%

#### Prominent thymus for age

- Was seen in 12/220 patients (about 5.45%).

### Associated other pulmonary and chest lesions

**Fig. 21:**
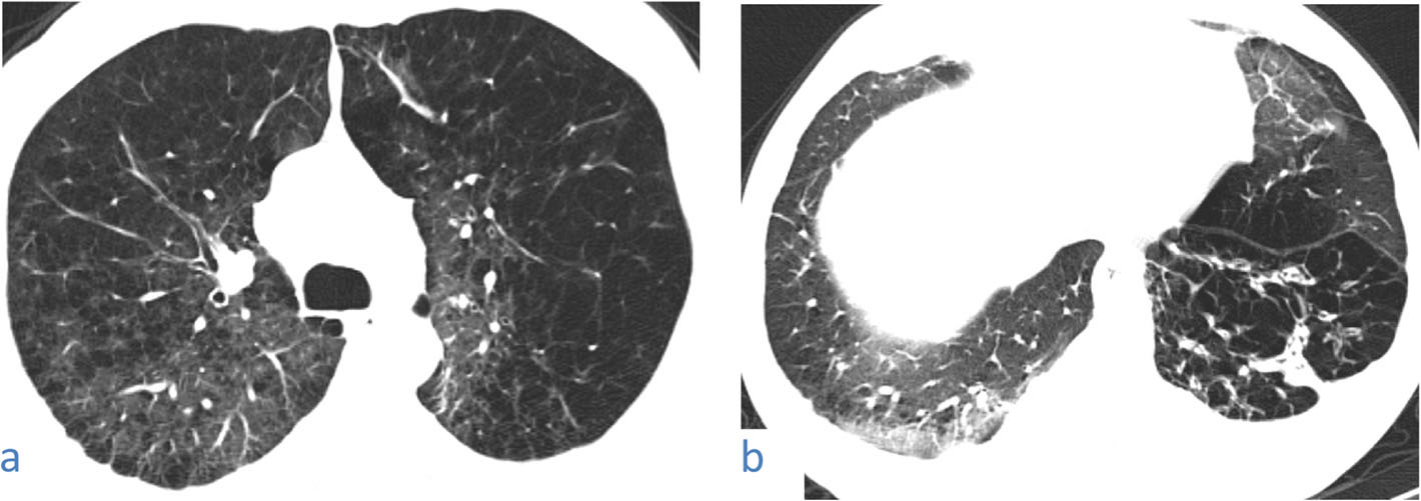
A 48-year-old male smoker with COVID-19. Axial CT image (**a**) shows marked upper lobes centrilobular emphysema with subpleural and patchy ground glass opacification (**b**) shows right lower lobe peripheral ground glass opacity. Vascular thickening is seen related to lesions. Left lower lobe shows architectural distortion with fibrous stripes

- Was seen in 7/220 patients (about 3.18%) (Fig. 21).

Table 6 shows a summary of associated other pulmonary and chest lesions.

**Table 6:** A summary of associated other pulmonary and chest lesions

Type of lesion	Number of patients	Percentage
Emphysema	3	1.36%
Sarcoidosis	1	0.45%
Subacute hypersensitivity pneumonitis	1	0.45%
Subcarinal bronchogenic cyst	1	0.45%
Asbestos exposure pleural changes	1	0.45%

In this study, we had three pediatric cases aged 10, 12, and 13 years old, the distribution and patterns of their lungs lesions did not differ from those of our adult patients.

## Assessing temporal changes

None of our patients had a follow-up study, however, in studying and analyzing the CT findings of all cases the following could be concluded as follows:

➢ Ground glass subpleural opacities were the earliest lesions encountered that could be seen as a single focus or multiple lesions. It was the only lesion noted in 63 cases (about 29%).

➢ Architectural distortion with any of its described lesions and reversed halo signs were the end stage findings. They were mostly lower lobar (78 cases [35.45%]) and bilateral (76 cases [34.54%]).

➢ Surfing the literature temporal changes of CT findings were described and suggested a pattern with consecutive appearance of lesions in COVID-19 patients; however, in our study, we met different patterns together in the same patient and even in the same lobe showing ground glass opacities, consolidation, crazy paving, and/ or architectural distortion in 78/220 patients (35.45%) (Figs. 12, 13, 14, 15, and 21).

➢ So actually temporal wise, we had early lesions with only ground glass opacities in 63 cases (about 29%), and late cases with only architectural distortion and reversed halo sign in 38 cases (17.27%), with 119 cases (54.09%) showing signs of early, progressive, peak, and absorptive stages all together.

➢ So regarding the temporal changes using our CT findings, we can suggest the following classification:

Stage 1: Only ground glass opacities detected. This corresponds to the early initial stage in literature.

Stage 2: In this stage, the disease progresses and the following can be encountered

i. Ground glass opacities together with other lesions.
ii. Crazy paving or consolidation alone or associated with other lesions.

Stage 3: Signs of architectural distortion and/or reversed halo sign not associated with other lesions. This corresponds to the absorptive stage in literature.

## Assessing severity

We applied the severity score described in the radiology assistant [7] but with a trivial modification for example instead of stating that the score is 1 or is 24, we wrote it as 1/25 or 24/25, respectively, which will reflect a mild case in the first condition and a severe one in the second, we found the results as such to be self-explanatory and does not require prior acquaintance with the scoring system for interpretation.

## Reporting

In our early experience with COVID-19 cases, only those with typical lesions according to the Radiological Society of North America (RSNA) released consensus statement [15] were diagnosed as such. While any other pattern of inflammation considered as atypical or indeterminate by the same consensus was described and reported accordingly. However, lately since the cases flow have peaked, any CT sign of inflammation was proved to be COVID-19 even cases previously described as atypical as for example lobar pneumonia, 2 of our late cases had only lobar pneumonia in CT chest and both were proved PCR positive. So actually recently any case with clinical suspicion and where CT shows signs of inflammation is considered as COVID-19 and is proved to be as such by laboratory assessments.

## Pitfalls

**Fig. 22:**
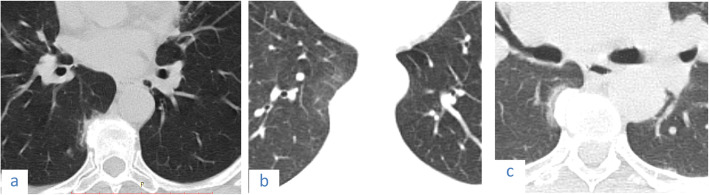
Three cases of osteophyte induced adjacent pulmonary fibrosis and atelectasis are seen as right lower lobe subpleural ground glass opacification and reticulations adjacent to a dorsal spine exuberant osteophyte in (**a**) 57-year-old female, (**b**) 63-year-old female, and (**c**) female patient 48 years old

Osteophyte induced adjacent pulmonary fibrosis and atelectasis is a lesion that may be mistaken for early subpleural ground glass opacification caused by COVID-19; however, the key to its diagnosis is the presence of exuberant osteophytes and the classic paravertebral position of the lesion (Fig. 22).

## Discussion

This cross-sectional multicenter study has been conducted on 220 Egyptian patients, 68 (30.9%) females and 152 (69.1 %) males, their age range was 10-92 years (average 49.198 years), from March to the end of May 2020. Non-contrast MSCT chest was done to all patients. Data assessment and analysis for lesions’ pattern, localization, and severity was done. Bilateral affection was seen in 168/220 cases (76.36%). Multilobar affection was noted in 186/220 cases (84.54%). Lower lobes affection was noted in 179/220 cases (81.36%). Peripheral/subpleural affection was noted in 203/220 cases (92.27%).The common (ground glass opacities, consolidation, crazy paving, vascular thickening, traction bronchiectasis, vacuolar sign, architectural distortion signs, and reversed halo sign) and the uncommon CT patterns (halo sign, masses, nodules, lobar affection, tree in-bud-pattern, and cysts) were discussed and associated extra-pulmonary lesions were described. Our results globally agree with the published literature [3–9, 11, 13, 14, 16–18].

In our study, we had only three pediatric patients; the CT findings were ground glass opacification in two cases and consolidation in one case. The lesions were similar to adult’s lesions in distribution. This complies with the findings described in literature [6].

A common pitfall that should be avoided in the diagnosis is mistaking osteophyte induced adjacent pulmonary fibrosis and atelectasis for subpleural COVID-19 lesions. The lesion is classically seen in the paravertebral region and is related to a vertebral osteophyte [19].

In the current situation where the numbers of cases of COVID-19 are in continuous ascent, we considered any CT pattern that reflects inflammation in a patient with clinical suspicion of COVID-19 as a case of COVID-19. Declaring that an inflammatory CT lesions is not COVID-19 because its pattern does not conform with the typical patterns of the disease is not logical in this time of peaking pandemic .Thus, applying the classification systems [6, 7, 10, 11] that actually depended on staging the probability of the disease (typical, atypical, or indeterminate) to any lesion in CT that suggests inflammation was not considered feasible by our team starting this May. According to our results, we regarded the lesions as either common to be seen with COVID-19 and thus stated as typical in the radiology report, or as uncommon lesions for COVID-19 (as nodules and masses [13], halo sign, lobar consolidation, or tree-in bud pattern) that if noted in a patient with clinical suspicion of the disease or a contact of a known case will be reported as an uncommon CT pattern of COVID-19, however, it reflects pulmonary infection thus COVID-19 should be considered.

Applying a severity score may be requested by clinicians. From our point of view, all the current severity scoring systems of COVID-19 [7, 20] have the following drawbacks:

➢ They are all based on visual assessment.

➢ They are totally subjective.

➢ They are time consuming.

➢ They are rather sophisticated.

➢ The clinician receiving the report needs to be acquainted with the scoring system you are using which is not very feasible if he is not in the same institute or in case of multicenter cases.

However, applying a scoring system may be needed for clinical purposes and in case of follow-up studies. We tried applying the severity score described in the radiology assistant [7] but with a trivial modification for example instead of stating that the score is 1 or is 24, we wrote it as 1/25 or 24/25, respectively, which will reflect a mild case in the first condition and a severe one in the second, we found that the results as such are self-explanatory and does not require prior acquaintance with the scoring system for interpretation.

In assessing the temporal changes of our cases, we had early lesions with only ground-glass opacities in 63cases (about 29%), and late cases with only architectural distortion and/or reversed halo sign in 38 cases (17.27%), with 119 cases (54.09%) showing signs of early, progressive, peak, and absorptive stages all together, thus we suggested a classification were stage 1 only have ground-glass opacities detected. This corresponds to the early initial stage in literature, stage 2 where the disease progresses may show ground-glass opacities with other lesions, crazy paving, or consolidation alone or associated with other lesions, and stage 3 showing signs of architectural distortion and/or reversed halo sign not associated with other lesions. This corresponds to the absorptive stage in literature [6].

In the early days of the COVID-19 crises, the role of CT chest in the management was debated both in China and later worldwide. Some authors have recommended it while other authors have restrained its use [16, 17]. The Fleischner Society has announced certain rules for the application of CT in cases of COVID-19 [6, 21], in April 2020, according to which CT could be done in cases of mild clinical features if patient is at risk for disease progression, also in patients with suspected COVID-19 who present with moderate-severe clinical features and a high pretest probability of disease, or there is worsening of the respiratory status. By the end of May 2020, in Egypt, those conditions are actually applicable and in the current pandemic peaking crisis according to our experience, CT is considered an essential cornerstone in the management of clinically suspected cases of COVID-19.

## Conclusion

CT plays a basic essential role in the diagnosing COVID-19 in the current declared pandemic.

## Data Availability

The datasets used and/or analyzed during the study are available upon reasonable request.

## Abbreviations

COVID-19: Coronavirus disease 2019
CO-RAD: COVID-19 reporting and data system
COVID-Rad: COVID-19 reporting and data system
CT: Computed tomography
FOV: Field of view
MDCT: Multidetector computed tomography
MSCT: Multislice computed tomography
RAD: Reporting and data system
RSNA: Radiological Society of North America
SARS-CoV-: Severe acute respiratory syndrome coronavirus 2 of the genus Beta-coronavirus that is the causative agent of COVID-19
SPSS: Statistical Package for the Social Science
